# Transcriptome analysis reveals the potential biological function of FSCN1 in HeLa cervical cancer cells

**DOI:** 10.7717/peerj.12909

**Published:** 2022-02-02

**Authors:** Fengqin Guo, Yanliang Liu, Yanxiang Cheng, Qifan Zhang, Weili Quan, Yaxun Wei, Li Hong

**Affiliations:** 1 Department of Obstetrics & Gynecology, Renmin Hospital of Wuhan University, Wuhan, Hubei Province, China; 2 Department of Gastrointestinal Surgery II, Renmin Hospital of Wuhan University, Wuhan, Hubei Province, China; 3 ABLife BioBigData Institute, Wuhan, Hubei Province, China; 4 Center for Genome Analysis, ABLife Inc., Wuhan, Hubei Province, China

**Keywords:** FSCN1, Gene knockdown, RNA-seq, Gene expression profile, Transcription regulation

## Abstract

Fascin actin-bundling protein 1 (FSCN1), an actin-bundling protein associated with cell migration and invasion, is highly expressed in various tumor tissues. FSCN1 has also been reported to be a marker of increased invasive potential in cervical cancers. However, the functions of FSCN1 are still not fully understood in cervical cancers. Here, the gene expression profile of HeLa cells transfected with FSCN1 shRNA (shFSCN1) was compared with that of cells transfected with empty vector (shCtrl). The results showed that shFSCN1 extensively affected the transcription level of 5,043 genes in HeLa cells. In particular, Gene Ontology (GO) analysis showed that a large number of upregulated genes were annotated with terms including transcription regulation and DNA binding. The downregulated genes were enriched in some cancer pathways, including angiogenesis and cell adhesion. qPCR validation confirmed that FSCN1 knockdown significantly affected the expression of selected genes in HeLa cells either negatively or positively. Expression analysis in TCGA (The Cancer Genome Atlas) revealed that FSCN1 had negative correlations with several transcription factors and a positive correlation with an angiogenic factor (angiopoietin like 4, *ANGPTL4*) in cervical tumor tissue. In particular, validation by Western blotting showed that FSCN1 knockdown decreased the protein level of ANGPTL4. Our results demonstrated that FSCN1 is not only an actin-binding protein but also a transcriptional regulator and an angiogenic factor in cervical cancer. Thus, our study provides important insights for further study on the regulatory mechanism of FSCN1 in cervical cancer.

## Introduction

Cervical cancer is one of the most common cancers among women, with more than half a million new cases and more than 300,000 deaths each year worldwide. The vast majority of cervical cancer cases are caused by infection with a high-risk subtype of human papillomavirus (HPV) (Li et al., 2019). However, due to the lack of effective screening and HPV vaccination programs, approximately 90% of cervical cancer cases worldwide occur in low-income and developing countries, particularly in remote rural areas. Based on the diagnosis of cervical cancer and the patient’s condition, surgical resection, chemotherapy and radiotherapy can achieve good therapeutic effects. For metastatic and recurrent cervical cancer, although bevacizumab can effectively prolong survival, the overall prognosis is still not ideal (Cohen et al., 2019). Therefore, it is still an urgent need to further study the molecular mechanism of cervical cancer and find specific diagnostic molecules and therapeutic targets.

The FSCN1 protein (Fascin actin—bundling protein 1), also called Fascin, is a member of the actin family , and plays an important role in processes such as cell migration, cell mobility and cell adhesion (Adams, 2004). FSCN1 is highly expressed in various tumor tissues (Adams, 2015), is involved in the formation of filopodia in tumor cells, and plays an important role in the initial stage of tumor cell migration and invasion (Li et al., 2018a). Understanding the molecular mechanism by which FSCN1 functions in tumor metastasis will aid in identifying a more suitable therapeutic target for metastatic cancer. On the one hand, a large number of studies have reported the regulatory mechanisms mediating abnormal expression of FSCN1 in cancer. Phosphorylated SP1 protein, as well as microRNAs, including miR-145, miR-133a, and miR-133b, and lncRNAs, including PVT1, ROR, and TTN-AS1, could contribute to regulating the expression of FSCN1 (Kano et al., 2010; Lu et al., 2010; Shang et al., 2018; Shen et al., 2019; Yu et al., 2017). These studies provide a basis for developing drugs targeting microRNAs and lncRNAs or screening phosphorylase inhibitors as anticancer drugs by determining their ability to regulate the expression of FSCN1. Alternatively, some studies have explored the possible mechanisms by which FSCN1 affects the occurrence and development of cancer. FSCN1 can regulate the expression of CTGF and CYR61 by downregulating the expression of the activating factor THBS1 in TGF-β pathway, thus affecting the proliferation and invasion of esophageal cancer cells (Xie et al., 2010). It was found that the phosphorylation level of the FSCN1 protein is correlated with the occurrence and development of esophageal cancer (Zeng et al., 2017; Zhao et al., 2010). In addition, the FSCN1 protein can localize in the nucleus and function as an epigenetic modulator of genes essential for amino acid metabolism (Saad et al., 2016). Given that FSCN1 also has important functions in normal cells, the currently developed targeted drugs for FSCN1 may also adversely affect the functions of normal cells. Therefore, it is necessary to continue to study the regulatory mechanism of FSCN1 in the expression of genes encoding proteins in its downstream signaling pathway, which will provide a scientific basis for identifying a more suitable therapeutic target.

In fact, a limited number of studies have reported the roles of FSCN1 in cervical cancer. There is significantly higher epithelial FSCN1 expression and microvessel count in high-grade squamous intraepithelial lesions than in cervical tissues with chronic inflammation (Hou et al., 2009; Kabukcuoglu et al., 2005), and these increases may be a marker of increased invasive potential in high-grade cervical intraepithelial neoplasia (Koay, Crook & Stewart, 2014). FSCN1 was found to be overexpressed in squamous cell carcinoma of the cervix and might be involved in the metastasis of cancers induced by some types of HPV, hypothetically through attenuation of intercellular adhesion and induction of cell motility (Yousefi Ghalejoogh et al., 2018). Knockdown of FSCN1 can inhibit the growth of CaSki cancer cells and reduce tumorigenicity in nude mice by regulating the expression of PCNA, survivin, CDK4 and p21 (Li et al., 2018b). FSCN1 expression levels were found to be negatively correlated with miR-145 expression levels in cervical cancer tissues, and overexpression of miR-145 or knockdown of FSCN1 dramatically inhibited the proliferation of HeLa cells (Ma & Li, 2019). However, the functions of FSCN1 in cervical cancers are still not fully understood. In particular, the genome-wide regulated target genes of FSCN1 in cervical cancer remain unclear.

Here, to further explore the regulated targets of FSCN1, we knocked down FSCN1 in HeLa cells isolated from cervical cancer tissue. Then, comprehensive gene expression profiles of FSCN1 knockdown cells and controls were detected by high-throughput RNA sequencing (RNA-Seq) to identify genome-wide targets regulated by FSCN1. The results showed that FSCN1 knockdown can extensively affect the expression of a large number of genes. In particular, FSCN1 promoted the expression of metastasis-associated genes and inhibited the expression of many transcription factors at the mRNA level in HeLa cells. These results indicated an unreported transcriptional regulatory role of FSCN1. Our study provides an important basis and data platform to further clarify the role of FSCN1 in mediating the metastasis of cervical cancer.

## Material and methods

### Cell culture and transfection

The human cervical cancer cell line HeLa was purchased from the Institute of Biochemistry and Cell Biology, Chinese Academy of Sciences (Shanghai, China). Cells were cultured in Dulbecco’s modified Eagle’s medium (DMEM) containing 10% fetal bovine serum (FBS). HeLa cells were transfected with the pGFP-B-RS vector with shRNA sequence (CCCTTGCCTTTCAAACTGGAA) inserted to knock down FSCN1. A vector without the shRNA was transfected into HeLa cells as the control. In addition, FSCN1 siRNA (CCCTTGCCTTTCAAACTGGAA) and nontargeting control siRNA (UUCUCCGAACGUGUCACGUTT) were used to transfect HeLa cells to knock down FSCN1. Both the FSCN1 shRNA and siRNA had the same sequence, which targeted exon 5 in the 3′ UTR of FSCN1. Transfection was performed *via* Lipofectamine 2000 (Invitrogen, Carlsbad, CA, USA) following the manufacturer’s protocol. After 48 h, transfected HeLa cells were collected for qPCR and Western blot analysis of FSCN1 expression.

### Assessment of knockdown efficiency

Total RNA was isolated from HeLa cells transfected with different vectors using TRIzol reagent (Ambion, USA). Then, cDNA was synthesized. qPCR was performed using Bestar SYBR Green RT-PCR Master Mix (DBI Bioscience, Shanghai, China) on a Bio-Rad S1000 thermal cycler. GAPDH (glyceraldehyde-3-phosphate dehydrogenase) was used as the reference gene to assess the efficiency of FSCN1 knockdown. The primers used for qPCR are listed in Table S1. The expression of FSCN1 was then normalized to GAPDH using the 2^−ΔΔCT^ method.

### Western blotting

HeLa cells were lysed using RIPA buffer (Beyotime) on ice for 30 min. Then, centrifugation of the sample was performed at 12,000 rpm at 4 °C for 10 min. The supernatant sample was separated by SDS–PAGE and transferred onto polyvinylidene fluoride membranes. The membranes were blocked using nonfat milk and incubated overnight with primary antibodies against FSCN1 (1:1,000, ABclonal), ANGPTL4 (1:1,000, ABclonal), and GAPDH (1:5,000, ATA). Subsequently, the membranes were incubated with secondary antibodies (Proteintech, Wuhan). Bound secondary antibody was detected using enhanced chemiluminescence (ECL) reagent (Bio-Rad, 170506).

### Library preparation and sequencing

Total RNA was extracted from HeLa cells with TRIzol (Ambion) and purified by two phenol–chloroform extractions. Then, the purified total RNA was treated with RQ1 DNase (Promega, Madison, WI, USA) to remove DNA. The quality and quantity of further purified RNA was assessed by measurement of the absorbance ratio at 260 nm/280 nm (A260/A280) using a Smartspec Plus spectrophotometer (BioRad, USA). Agarose gel electrophoresis (1.5%) was performed to assess the integrity of the purified RNA.

For RNA-seq library preparation, 1 µg RNA was used for each sample. Oligo(dT)-conjugated magnetic beads (Invitrogen, Carlsbad, CA, USA) were used to purify and concentrate polyA mRNAs from the total RNA sample. RNA in the purified and concentrated sample was fragmented. End-repair and 5′ adaptor ligation of fragmented RNA were performed. Then, the RNA was reverse transcribed using primers harboring a 3′ adaptor sequence and randomized hexamers for cDNA. The cDNAs were further amplified and purified. The purified cDNAs were stored at −80 °C until sequencing.

High-throughput sequencing libraries were prepared according to the manufacturer’s instructions. Paired-end sequencing (151-bp) was conducted with the Illumina HiSeq4000 system at a commercial company (ABLife Inc., Wuhan, China).

### Clean and alignment of raw sequencing data

First, raw sequencing reads with more than 2-N bases were discarded. Then, FASTX-Toolkit (Version 0.0.13) was used to trim the adaptors and low-quality bases from the raw reads. The resulting reads less than 16 nt were further discarded. TopHat2 was used to align the clean reads to the GRch38 genome allowing four mismatches (Li et al., 2013). Then, the uniquely mapped reads for each gene were used to calculate the FPKM (paired-end fragments per kilobase of exon per million fragments mapped) values (Trapnell et al., 2010).

### Differentially Expressed Genes (DEG) analysis

FPKM was used to evaluate the expression level of genes. edgeR software was used to identify the differentially expressed genes (DEGs) (Robinson, McCarthy & Smyth, 2010) with fold change threshold (fold change ≥2 or ≤0.5) and a false discovery rate threshold (FDR < 0.05).

### Functional enrichment analysis of DEGs

Gene Ontology (GO) analysis was used to predict the gene function and calculate the functional category distribution frequency, which annotates an input set of genes with putative pathways and disease relationships based on mapping to genes with known annotations (Xie et al., 2011). A hypergeometric test with Benjamini–Hochberg FDR correction were used to define the enrichment of each pathway (corrected *p* value < 0.05).

### Validation of DEG expression by qPCR

Quantitative real-time PCR (qPCR) of selected DEGs was conducted to elucidate the validity of the RNA-seq data. GAPDH was used as a reference gene. Primers for qPCR are presented in Table S1. qPCR was conducted on the same RNA samples that were used for RNA-seq. The thermal cycling conditions for PCR were as follows: denaturation for 10 min at 95 °C, followed by 40 cycles of including denaturation for 15 s at 95 °C and annealing and extension for 1 min at 60 °C. Three technical replicates were performed for PCR amplification of each sample.

### Analysis of TCGA (The Cancer Genome Atlas) data

The RNA-seq data of cervical cancer in the TCGA database were downloaded from UCSC Xena (https://xenabrowser.net/datapages/). The expression levels (FPKM values) of FSCN1 and FSCN1-regulated DEGs in cervical cancer samples were analyzed. Kaplan–Meier (KM) analysis was used to estimate the survival rate, and the survival curve was plotted with the log-rank test to calculate the *P* value.

### Statistical analysis

Statistical analysis was performed using R (v3.1.3). qPCR data of the two groups were compared by an unpaired two-tailed t test at *P* values < 0.05. The qPCR data are presented as the mean  ± standard deviation (SD). Pearson correlation analysis was used to assess the correlation between the expression of FSCN1 and FSCN1-regulated DEGs in cervical cancer tissues from TCGA at *P* values < 0.05.

## Results

### FSCN1 was overexpressed in cervical cancer tissues and associated with poor prognosis

**Figure 1:**
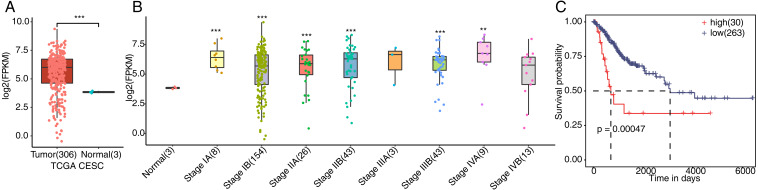
Expression of FSCN1 in clinical samples of cervical cancer in TCGA. (A) FSCN1 had high expression at the mRNA level in tumor tissue, based on TCGA samples. (B) FSCN1 had relatively high expression in samples of cervical cancer at different stages. (C) Patients with high expression (high) of FSCN1 had a lower survival rate than patients with low expression (low). Student’s *t* test was performed to compare FSCN1 expression in normal and tumor tissue, with significance considered to be indicated by a *P* value of less than 0.05. Kaplan–Meier (KM) analysis was used to estimate the survival rate and plot the survival curve, and the log-rank test was used to calculate the *P* value. The high and low FSCN1 expression groups were automatically separated by the KM analysis software. The number of samples in each group is indicated in parentheses. **P < 0.01; ***P < 0.001.

To verify whether FSCN1 was overexpressed in cervical cancer tissues, we detected the expression of FSCN1 in cervical cancer. We first analyzed the expression of FSCN1 in cervical cancer samples from the TCGA database, which includes the gene expression profile (FPKM) of 306 cervical cancer tumor and 3 normal tissues. The results showed that the expression of FSCN1 was obviously upregulated in cervical cancer tumor tissues compared to normal tissues (Fig. 1A). Moreover, FSCN1 showed relatively high expression in samples of cervical cancer at different stages of development compared to normal tissues (Fig. 1B). Additionally, the expression of FSCN1 was correlated with the prognosis of patients with cervical cancer. Compared to patients with low expression of FSCN1, patients with high expression had shorter overall survival times (Fig. 1C). Thus, FSCN1 might be a potential prognostic factor in cervical cancer.

### FSCN1 knockdown broadly affects the gene expression profile of HeLa cells

**Figure 2:**
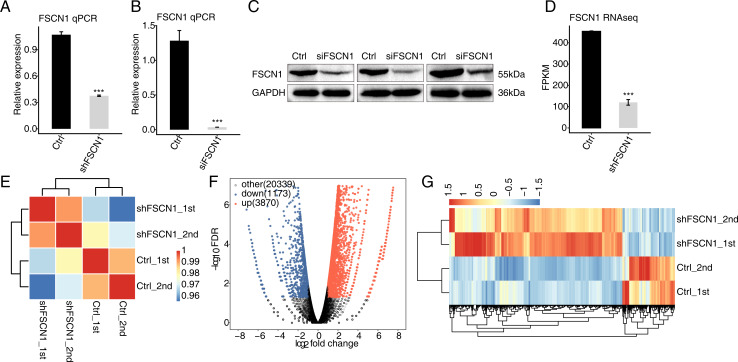
Effects of FSCN1 knockdown on the gene expression profile of HeLa cells. (A) FSCN1 knockdown by transfection with empty vector (Ctrl) or with FSCN1 shRNA (shFSCN1) was validated by qPCR. (B) FSCN1 knockdown by transfection with nontargeting siRNA or FSCN1 siRNA was validated by qPCR. (C) FSCN1 knockdown by transfection with nontargeting siRNA (Ctrl) or FSCN1 siRNA (siFSCN1) was validated by Western blotting. (D) FSCN1 knockdown was validated by RNA-seq. (E) Heatmap of the Pearson correlation analysis results showed similarity between control and FSCN1 knockdown samples. The correlation was calculated by transcript expression values of all expressed genes. (F) Differentially expressed genes (DEGs) were identified after FSCN1 knockdown. Red and blue dots represent upregulated and downregulated genes in the volcano plot, respectively. (G) Heatmap and hierarchical clustering of DEGs between control and FSCN1 knockdown samples. FPKM (paired-end fragments per kilobase of exon per million fragments mapped) values for each gene were log2-transformed and then median-centered. For qPCR, GAPDH was used as the reference gene. Student’s *t* test was performed to compare FSCN1-KD and control cells, with significance considered to be indicated by a *P* value of less than 0.05. ***P < 0.001.

To explore the targets regulated by FSCN1 in cervical cancer, the expression of FSCN1 was knocked down in HeLa cells by transfection of FSCN1 shRNA (shFSCN1). Compared with empty vector (shCtrl), FSCN1-shRNA effectively reduced the mRNA expression of FSCN1 (Fig. 2A). To further assess the transfection efficiency, we also knocked down FSCN1 with a siRNA having the same target sequence and a nontargeting siRNA. The results showed that this FSCN1 siRNA decreased the expression of FSCN1 at the mRNA and protein levels (Figs. 2B and 2C).

Then, RNA-seq was used to detect the gene expression profiles of FSCN1 knockdown and control HeLa cells. Both shFSCN1 and control HeLa cells had two biological replicates. There were four RNA-seq samples (shFSCN1_1st, shFSCN1_2nd, shCtrl_1st, and shCtrl_2nd). After cleaning the raw sequencing data, at least 73.4 million paired-end reads were obtained for each sample. After mapping of those clean reads to the human genome, more than 57.0 million uniquely mapped reads of each sample were used for gene expression determination and further gene expression analysis (Table S2).

The FPKM value was calculated to represent the expression level of each gene. The results showed that there were 25, 296 genes with FPKM >0 and 13,415 genes with FPKM > 1 in at least one sample (Tables S3 and Table S4). FSCN1 knockdown was further verified by the FPKM values of this gene in HeLa cells (Fig. 2D). Based on the FPKM values of each expressed gene, a correlation matrix was constructed for the four samples that were used for unsupervised hierarchical clustering. As shown, there was a clear separation of the shFSCN1 and control samples, with the two biological replicates clustered together (Fig. 2E). This result demonstrated that FSCN1 knockdown obviously changed the gene expression profile of HeLa cells.

Then, DEGs between the shFSCN1 and control cells were identified by edgeR to further compare the gene expression profile. A total of 3,870 upregulated and 1,173 downregulated DEGs were identified between shFSCN1 and control cells (Fig. 2F). All the DEGs are listed with detailed information including FPKM and fold change values (Table S5). In addition, there was consistent expression of these DEGs in both replicates of the shFSCN1 and control samples (Fig. 2G). These results showed that FSCN1 extensively regulates gene expression in HeLa cells.

### Functional analysis of genes upregulated and downregulated by FSCN1 knockdown in HeLa cells

**Figure 3:**
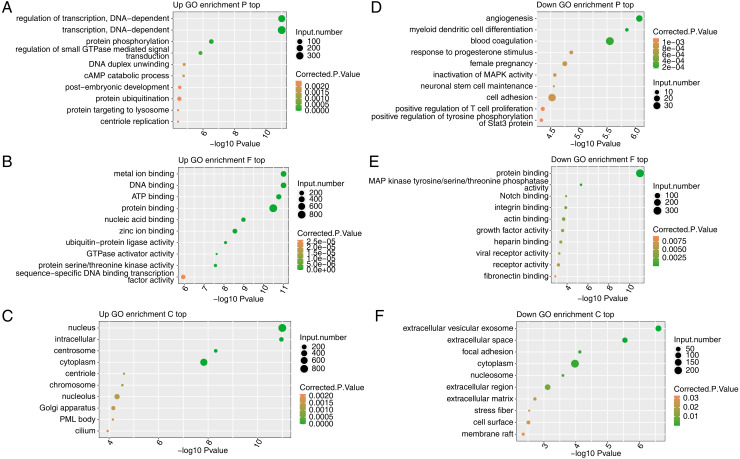
Functional analysis of DEGs after FSCN1 knockdown in HeLa cells. (A) The top 10 GO (Gene Ontology) biological process terms (GO enrichment P top) enriched with upregulated genes (top) after FSCN1 knockdown. (B) The top 10 GO molecular function terms (GO enrichment F top) enriched with upregulated genes after FSCN1 knockdown. (C) The top 10 GO cellular component terms (GO enrichment C top) enriched with upregulated genes after FSCN1 knockdown. (D) The top 10 GO biological process terms enriched with downregulated genes (down) after FSCN1 knockdown. (E) The top 10 GO molecular function terms enriched with downregulated genes after FSCN1 knockdown. (F) The top 10 GO cellular component terms enriched with downregulated genes after FSCN1 knockdown.

To reveal the potential roles of the DEGs, GO analysis was performed to annotate the 3,870 upregulated DEGs. As an input set of genes, these DEGs were annotated with putative pathways and disease relationships based on mapping to genes with known annotations. The GO analysis was divided into three categories: biological process (GO-P), molecular function (GO-F) and cellular component (GO-C). The results revealed 1,859, 1,859, and 2,124 upregulated genes annotated with 641 GO-P, 237 GO-F, and 186 GO-C terms, respectively (Table S6). The top 10 GO-P terms included DNA-dependent transcription and regulation of transcription (Fig. 3A). The top 10 GO-F terms included DNA binding and nucleic acid binding (Fig. 3B). Moreover, the top 10 GO-C terms included nucleus, chromosome and nucleolus (Fig. 3C). These results indicated that FSCN1 knockdown increased the expression of many transcription factors, which are distributed in the nucleus and can bind DNA.

To further reveal the roles of downregulated DEGs, GO analysis was performed to annotate those DEGs. The results revealed 561, 552, and 707 downregulated genes annotated with 245 GO-P, 74 GO-F, and 77 GO-C terms, respectively (Table S7). The top 10 GO-P terms included angiogenesis and cell adhesion (Fig. 3D). The top 10 GO-F terms included integrin binding and actin binding (Fig. 3E). Moreover, the top 10 GO-C terms included extracellular space and focal adhesion (Fig. 3F). These results indicated that FSCN1 positively regulates the expression of many genes associated with angiogenesis and cell adhesion.

### Validation of FSCN1-regulated genes in HeLa cells and cervical cancer tissues

**Figure 4:**
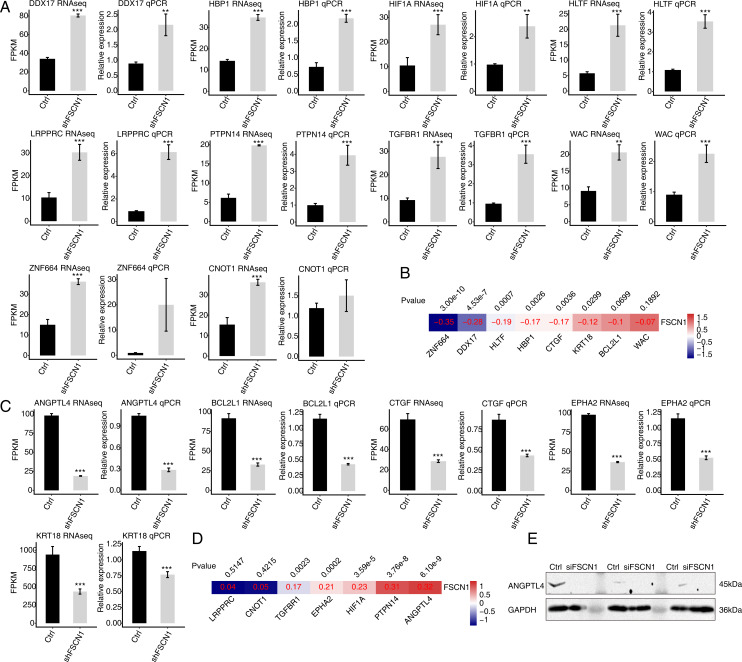
Validation of FSCN1-regulated genes in HeLa cells and cervical cancer tissues. (A) Expression levels of ten DEGs negatively regulated by *FSCN1* in FSCN1 knockdown (shFSCN1) and control cells (Ctrl) determined by RNA-seq (FPKM) (left) and qPCR (right). (B) Nine DEGs showed a negative correlation with *FSCN1* in 306 clinical samples of cervical cancer from the TCGA database. (C) Relative expression levels of five DEGs positively regulated by *FSCN1* in FSCN1 knockdown (shFSCN1) and control cells (Ctrl) determined by RNA-seq (FPKM) (left) and qPCR (right). (D) Six DEGs showed a positive correlation with *FSCN1* in 306 clinical samples of cervical cancer from the TCGA database. (D) The protein level of ANGPTL4 was detected by Western blotting in HeLa cells transfected with a vector containing nontargeting siRNA (Ctrl) or FSCN1 siRNA (siFSCN1). For qPCR, GAPDH was used as the reference gene. Student’s *t* test was performed to compare FSCN1 knockdown and control cells, with significance considered to be indicated by a *P* value of less than 0.05. **P < 0.01, ***P < 0.001; Pearson correlation coefficients for the relationship between FSCN1 expression and the expression of its regulated DEGs in cervical cancer samples was calculated at *P* values < 0.05.

To verify the effect of FSCN1 knockdown on the expression of these DEGs, qPCR was conducted to quantify the changes in the mRNA levels of these genes after FSCN1 knockdown in HeLa cells. Fifteen DEGs were selected for qPCR analysis, namely ten upregulated transcription factors—HBP1 (HMG-box transcription factor 1), HIF1A (hypoxia inducible factor 1, alpha subunit), HLTF (helicase-like transcription factor), LRPPRC (leucine-rich pentatricopeptide repeat containing), TGFBR1 (transforming growth factor, beta receptor 1), DDX17 (DEAD box helicase 17), CNOT1 (CCR4-NOT transcription complex, subunit 10), ZNF664 (zinc finger protein 664), WAC (WW domain containing adaptor with coiled-coil), and PTPN14 (protein tyrosine phosphatase, non-receptor type 14)—and five downregulated genes—KRT18 (keratin 18, type I), BCL2L1 (BCL2-like 1), ANGPTL4, CTGF (connective tissue growth factor), EPHA2 (EPH receptor A2). All selected DEGs had an FPKM value > 1 in at least one sample. The results showed that 13 of the 15 selected DEGs showed a significant increase or decrease after FSCN1 knockdown in HeLa cells, which was in agreement with the RNA-seq analysis (Figs. 4A and 4C). Although there was no significant difference in the qPCR results for ZNF664 and CNOT1, the trend was the same between the qPCR and RNA-seq data (Fig. 4A). These results indicated that FSCN1 negatively regulates the expression of HBP1, HIF1A, HLTF, LRPPRC, TGFBR1, DDX17, CNOT1, ZNF664, WAC, and PTPN14 (Fig. 4A), but positively regulates the expression of KRT18, BCL2L1, ANGPTL4, CTGF, and EPHA2 (Fig. 4C) in HeLa cells.

Then, we analyzed the correlations between the expression levels of FSCN1 and these genes in cervical cancer tissues from TCGA. The results showed that the expression of FSCN1 had a significant negative correlation (*P* < 0.05) with that of HLTF, HBP1, ZNF664, CTGF, DDX17, and KRT18 in cervical cancer tissues (Fig. 4B). There were significantly positive correlations (*P* < 0.05) between FSCN1 expression and the expression of PTPN14, TGFBR1, EPHA2, HIF1A and ANGPTL4 (Fig. 4D).

Thus, FSCN1 negatively regulates four transcription factors (HLTF, HBP1, ZNF664, DDX17) and positively regulates ANGPTL4 (an angiogenic factor) and EPHA2 in both HeLa cells and cervical cancer tissues (Fig. 4). Then, we further detected the effect of FSCN1 knockdown on the protein expression level of one selected target in HeLa cells. Western blotting showed that FSCN1 knockdown decreased the protein level of ANGPTL4 in HeLa cells (Fig. 4E). Altogether, these results indicated that FSCN1 is not only an actin binding protein, but also a transcriptional regulator and an angiogenic factor in cervical cancer.

## Discussion

Cervical cancer is a deadly malignant tumor worldwide, with an overall 5-year survival rate of less than 20% (Cohen et al., 2019). FSCN1 is an important mediator of carcinoma invasion and metastasis (Adams, 2004; Li et al., 2018a). In fact, FSCN1 is overexpressed in cervical cancer (Kabukcuoglu et al., 2005; Koay, Crook & Stewart, 2014; Yousefi Ghalejoogh et al., 2018), and FSCN1 knockdown inhibits the proliferation of CaSki and HeLa cervical cancer cells (Li et al., 2018b; Ma & Li, 2019). However, the functions of FSCN1 in cervical cancer are still not fully understood. In our study, FSCN1 knockdown was found to promote the expression of a large number of transcription factors and inhibit the expression of many genes associated with angiogenesis and cell adhesion at the transcriptional level in HeLa cells. The results indicated that FSCN1 is a transcriptional repressor and promotes metastasis in cervical cancer. Our study provides important cues for further study on the regulatory mechanism of FSCN1 in cervical cancer.

As reported, FSCN1 is highly expressed in many types of tumor tissue (Li et al., 2018a). Moreover, the expression of FSCN1 is higher in squamous intraepithelial lesions than cervical tissues (Kabukcuoglu et al., 2005; Koay, Crook & Stewart, 2014). Here, we downloaded and reanalyzed the expression of FSCN1 in all tumor and normal cervical tissues of TCGA, which also showed high expression of FSCN1 in tumor cervical tissues. In particular, high FSCN1 expression is associated with poor prognosis. Thus, it is important to study the underlying mechanisms of FSCN1 functions in cervical cancer.

In fact, a study showed that FSCN1 knockdown can inhibit the growth of CaSki cells by regulating the expression of PCNA, survivin, CDK4 and p21 (Li et al., 2018b). Here, we knocked down FSCN1 in HeLa cells and explored the genome-wide targets regulated by FSCN1 by transcriptome analysis. Our results showed that FSCN1 knockdown affects the expression of up to 5,043 genes, with 3,870 upregulated and 1,173 downregulated genes. These results indicated that FSCN1 affects the expression of a large number of genes in HeLa cells. A study showed that FSCN1 knockdown affected the expression of only 296 genes in esophageal squamous cancer cells (Xie et al., 2010). According to GO enrichment analysis of FSCN1-regulated genes in HeLa cells, FSCN1 knockdown decreases the expression of genes associated with cell adhesion, which is consistent with the results of previous studies showing that FSCN1 positively regulates the expression of cell adhesion genes (Anton et al., 2017; Misiura et al., 2020; Routray et al., 2017). In fact, cell adhesion molecules integrate cytoskeletal dynamics and cellular tension, which plays an important role in the metastasis of cancer cells (Parsons, Horwitz & Schwartz, 2010; Smart, Oleksak & Hartsough, 2021). Notably, FSCN1 knockdown increased the expression of many transcription factors in HeLa cells. Our study first demonstrated that FSCN1 may play a role as transcriptional regulator in a cell line. Thus, future work could be conducted to explore whether FSCN1 directly or indirectly binds the DNA of these transcription factors to regulate their transcription.

In our study, both the RNA-seq and qPCR results showed that FSCN1 regulates the expression of all fifteen selected DEGs in HeLa cells either negatively or positively. However, the expression of only six genes showed negative (four transcription factors, HLTF, HBP1, ZNF664, and DDX17) or positive (ANGPTL4 and EPHA2) expression correlations with FSCN1 expression in cervical tumor tissue from TCGA, which is consistent with the direction of regulation by FSCN1 observed in HeLa cells. Our explanation is that cancer tissues exhibit cellular heterogeneity, and some genes may show relatively high expression in certain cell types, as has been found by single-cell RNA sequencing of cancer tissue (Suva & Tirosh, 2019; Zhang et al., 2016). For example, HBP1 is a transcription factor of the HMG box family and functions as a tumor suppressor, which showed lower expression in several tumor types relative to matched normal tissues (Bollaert, De Rocca Serra & Demoulin, 2019). HBP1 could be downregulated by growth factors *via* the PI3K/PKB/FOXO pathway in breast cancer (Coomans De Brachène et al., 2014). Our results indicated that FSCN1 is overexpressed in cervical cancer tissue and negatively regulates the expression of HBP1 in HeLa cells. In addition, ANGPTL4 is overexpressed in cervical cancer, which predicts poor prognosis (Nie et al., 2019). ANGPTL4 knockdown inhibits proliferation and promotes apoptosis in SiHa cervical cancer cells (Nie et al., 2016). Our results showed that FSCN1 positively regulates the expression of ANGPTL4 in HeLa cells. It could be speculated that both HBP1 and ANGPTL4 are important regulated targets of FSCN1 in cervical cancer. Therefore, further study should be conducted to reveal the molecular mechanism by which FSCN1 regulates the expression of HBP1 and ANGPTL4 in cervical cancer.

## Conclusions

In summary, our study showed that FSCN1 is overexpressed in cervical cancer tissue and that genome-wide FSCN1 knockdown regulates the expression of target genes in HeLa cells. It is not surprising that FSCN1 promotes the expression of many genes associated with angiogenesis and cell adhesion in HeLa cells. However, we first revealed that FSCN1 inhibits the expression of a large number of transcription factors in HeLa cells. These results indicate that FSCN1 may be a transcriptional regulator in cervical cancer. Thus, FSCN1 may functions in the development of cancer *via* a variety of mechanisms. Our study provides important cues for further study on the regulatory mechanism of FSCN1 in cervical cancer.

## Supplemental Information

10.7717/peerj.12909/supp-1 Table S1 Primers that were used for qPCR

10.7717/peerj.12909/supp-2 Table S2 Summary of sample names, description, the RNA-seq sequencing information and mapping results in each sample

10.7717/peerj.12909/supp-3 Table S3 Gene expression level (FPKM)

10.7717/peerj.12909/supp-4 Table S4 Human genes detected by different FPKM cut off

10.7717/peerj.12909/supp-5 Table S5 Differential expression of genes between FSCN1-KD and control

10.7717/peerj.12909/supp-6 Table S6 GO terms of upregulated genes after FSCN1-KD

10.7717/peerj.12909/supp-7 Table S7 GO terms of downregulated genes after FSCN1-KD

10.7717/peerj.12909/supp-8 Data S1 Raw data for qPCR validation of DEG

10.7717/peerj.12909/supp-9 Data S2 Raw data for Western Blotting

## Additional Information and Declarations

### Data Availability

The following information was supplied regarding data availability: All data generated or analyzed during this study have been included in this published article and its Supplementary Information Files. The raw data of RNA-seq are available in the NCBI Gene Expression Omnibus and are accessible through GEO series accession number GSE149450.
