# Supplementary material for: Transcriptome analysis reveals the potential biological function of FSCN1 in HeLa cervical cancer cells
Source: PeerJ. 2022 Feb 2;10:e12909. doi: 10.7717/peerj.12909 (PMC8817631; doi:10.7717/peerj.12909)
Supplement: Data S2 [file peerj-10-12909-s009.docx]

Photograph of FSCN1 band (replicate 1)





Photograph of GAPDH band (replicate 1)





Photograph of FSCN1 band (replicate 2)





Photograph of GAPDH band (replicate 2)





Photograph of FSCN1 band (replicate 3)





Photograph of GAPDH band (replicate 3)


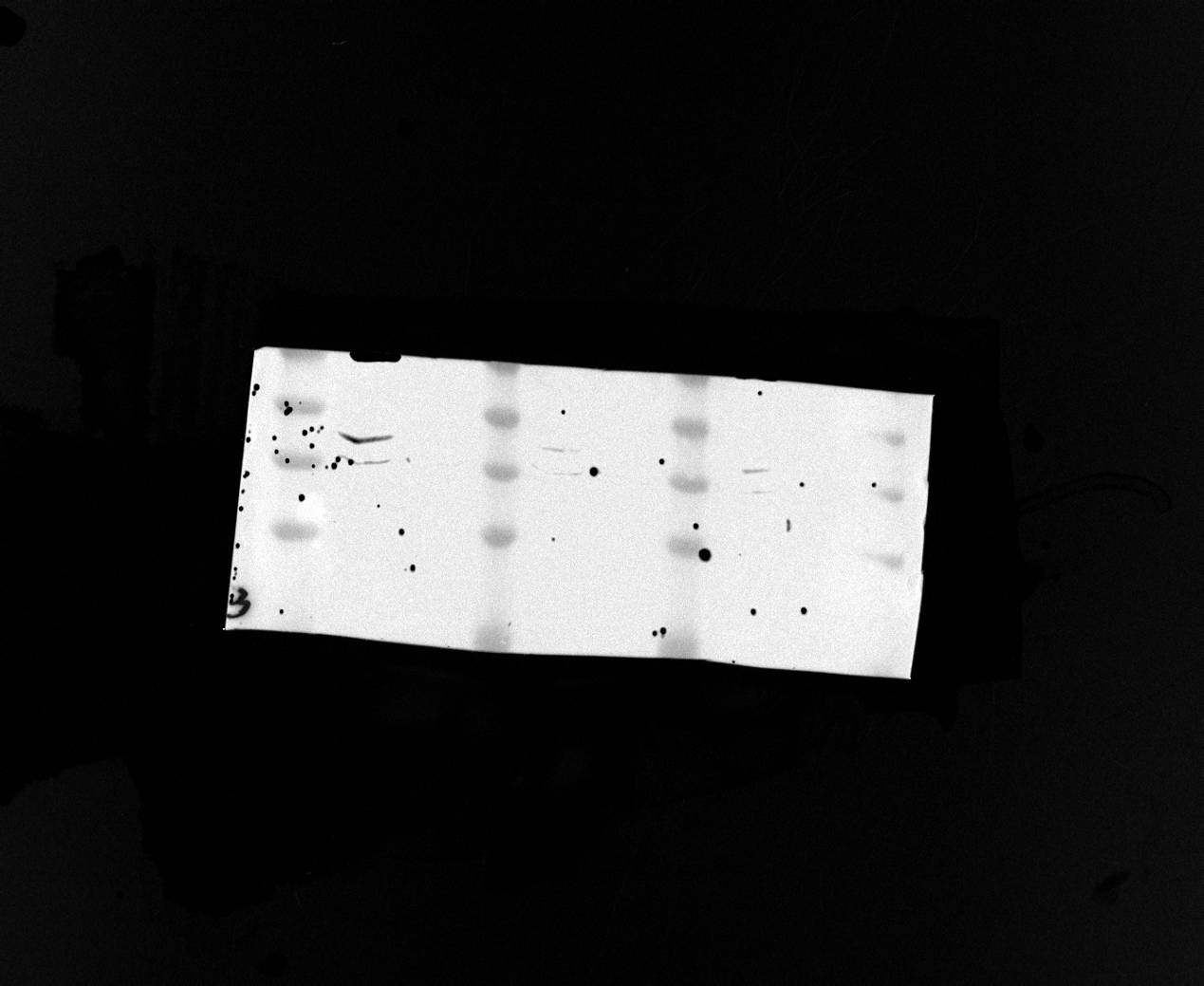


Photograph of ANGPTL4 band (replicate 3)


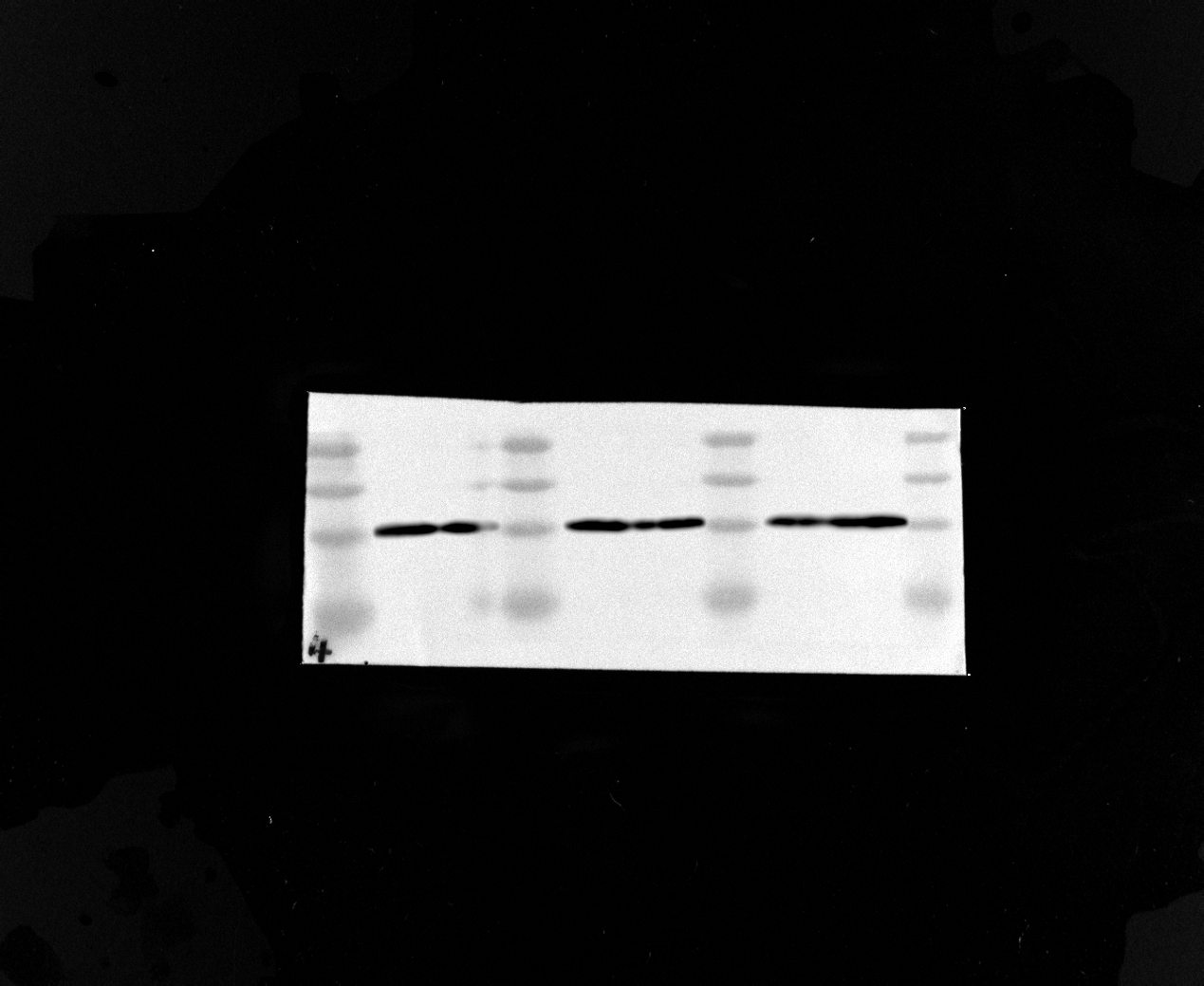


Photograph of GAPDH band
